# Molecular Genetic Markers in Female Reproductive Cancers

**DOI:** 10.1155/2010/307460

**Published:** 2011-04-18

**Authors:** Tian-Li Wang, Ben Davidson, Phillip J. Buckhaults, Chiun-Sheng Huang, Gudi Poul

**Affiliations:** ^1^Departments of Gynecology and Obstetrics and Oncology, Johns Hopkins University School of Medicine, Baltimore, MD 21231, USA; ^2^Department of Pathology, Norwegian Radium Hospital, Rikshospitalet University Hospital, Montebello, 0310 Oslo, Norway; ^3^Departments of Pathology and Microbiology and Immunology, The University of South Carolina School of Medicine, Columbia, SC 29203, USA; ^4^Department of Medicine, National Taiwan University, Taipei 10617, Taiwan; ^5^Department of Internal Medicine I, Center for Hematology and Oncology, Wilhelminenspital, 1160 Vienna, Austria

Cancer is a complex genetic disease as a result of accumulated genomic alterations which serve as the driving force in initiating tumor development and propelling tumor progression. Recent advances in molecular genomic technology and the success of human genome project have empowered investigators with new tools in analyzing cancer genome and transcriptome in great details and have expedited the discovery of new cancer-associated genes. Decoding the genetic history present in tumor DNA, as well as identification and characterization of molecular changes involving cancer-associated genes and the pathways they controlled, has not only shed new light on the molecular etiology of cancer, but also has promises for the development of new diagnostic markers and novel therapeutics. In this special issue, the papers focus on recent findings of markers identified in female reproductive cancers including breast, ovarian, endometrial, and trophoblast tumors. The selected papers are not an exhaustive representation of the area of biomarkers in female reproductive cancers. Nonetheless, they represent the rich and many-faceted knowledge that we have the pleasure of sharing that with readers. We would like to thank the authors for their excellent contributions to this special issue and the reviewers' insightful input to ensure the quality of this special issue. 

Of particularly interest are the two timely review articles by A. M. Karst et al. and A. L. Gross et al. focusing on the possible precursor lesions in the development of female reproductive cancer. The tubal origin of “ovarian” cancer has recently been in the research spot-light because of the identification of potential precursor lesions of ovarian cancer, termed “serous tubal intraepithelial carcinoma” in the fallopian tubes. Both articles critically review the published studies and provide unique perspectives of the impact and significance of this new hypothesis in the field of cancer research. 

Review articles focusing on biomarkers including genetic (K. M. Hirshfield et al.), microRNA (H. M. Heneghan et al.), and cytokine (F. Barbieri et al.) summarize recent advances in biomarker discovery and the translational application. Those articles will serve as the source for the investigators who wish to pursue in evaluating new potential markers in the female reproductive cancer research. They also present the new challenges in biomarker discovery, validation, and verification. 

In addition to the five review articles, there are several exciting original research articles describing discovery and validation of biomarkers in female reproductive cancers. Those papers include the studies of fatty acid synthase (S. M. Ueda et al.), miR200c (D. R. Cochrane et al.), gene expression (S. Konstantinovsky et al.), and cytogenetic markers (F. Micci et al.). The findings from those studies could serve as biological foundation for future exploration of how those markers contribute to disease progression. The biomarkers identified in these studies could also serve as a road map for development of novel diagnosis tools and new targeted therapies. This issue also reports a synergistic effect of Pten loss and oncogenic Kras mutation on the endometrial cancer development (I. H. Kim et al.) and the result provides new insight into the pathogenesis of endometrial carcinoma. Finally, K. L. Yap et al. applied molecular genetic markers to demonstrate a lack of a Y-chromosomal complement in the majority of gestational trophoblastic neoplasms, suggesting that most trophoblastic tumors are derived from previous molar gestations. Because the molecular characters in gestational trophoblastic disease are understudied, this short report should provide new insight into the pathogenesis of this rare but highly interesting gynecological neoplasm. 

In conclusion, this special issue is by no means an attempt to be comprehensive in covering the field of female reproductive cancer, but it provides excellent reviews and research articles that may be useful for readers in this field. We hope that this special issue will inspire investigators in launching new research endeavor in this field and are grateful for the opportunity to manage this special issue.

*Tian-Li Wang* *Tian-Li Wang* *Ben Davidson* *Ben Davidson* *Phillip J. Buckhaults* *Phillip J. Buckhaults* *Chiun-Sheng Huang* *Chiun-Sheng Huang* *Gudi Poul* *Gudi Poul*

